# The genome-wide effects of ionizing radiation on mutation induction in the mammalian germline

**DOI:** 10.1038/ncomms7684

**Published:** 2015-03-26

**Authors:** Adeolu B. Adewoye, Sarah J. Lindsay, Yuri E. Dubrova, Matthew E. Hurles

**Affiliations:** 1 Department of Genetics, University of Leicester, Leicester LE1 7RH, UK; 2 Wellcome Trust Sanger Institute, Wellcome Trust Genome Campus, Hinxton, Cambridge CB10 1SA, UK

## Abstract

The ability to predict the genetic consequences of human exposure to ionizing radiation has been a long-standing goal of human genetics in the past 50 years. Here we present the results of an unbiased, comprehensive genome-wide survey of the range of germline mutations induced in laboratory mice after parental exposure to ionizing radiation and show irradiation markedly alters the frequency and spectrum of *de novo* mutations. Here we show that the frequency of *de novo* copy number variants (CNVs) and insertion/deletion events (indels) is significantly elevated in offspring of exposed fathers. We also show that the spectrum of induced *de novo* single-nucleotide variants (SNVs) is strikingly different; with clustered mutations being significantly over-represented in the offspring of irradiated males. Our study highlights the specific classes of radiation-induced DNA lesions that evade repair and result in germline mutation and paves the way for similarly comprehensive characterizations of other germline mutagens.

## Abstract (web summary)

Ionizing radiation (IR) is an extensively studied mutagenic agent that can lead to the accumulation of extra mutations in the offspring of irradiated parents. Here the authors provide a comprehensive genome-wide survey of the consequences of IR on the mammalian germline.

## Graphical abstract

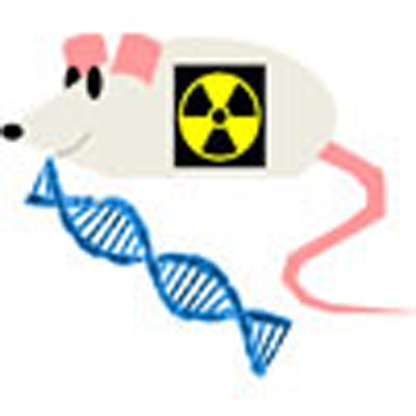

Ionizing radiation (IR) is an extensively studied mutagenic agent, exposure to which results in different types of DNA damage, ranging from modified nucleotides to double-stand breaks^1^. The mutagenic effects of IR on the germline are of particular concern as they lead to the accumulation of extra mutations in the offspring of irradiated parents^2^. Despite numerous efforts^3^, little is known about the genetic effects of radiation exposure in humans and the only definitive evidence for germline mutation induction *in vivo* in mammals comes from mouse studies^2^,^4^. Moreover, the current estimates of the germline genetic hazards of radiation and other mutagens for humans have been derived from the results obtained from the analysis of only a handful of protein-coding genes (typically seven)^4^,^5^. Although the results of these studies provide important information regarding the pattern of mutation induction in the germline of irradiated parents, the analysis of just seven protein-coding genes cannot accurately predict the genome-wide pattern of mutation induction. The lack of genome-wide data describing the pattern of mutation induction by IR or other mutagens also precludes further extrapolation from the results of mouse studies on the predicted excess of genetic syndromes among the offspring of exposed parents. Recent advances in genetic technologies have provided new microarray-based and next-generation sequencing-based tools for the genome-wide analysis of genetic variation, which have the potential for characterizing germline mutation in humans and mice. Here we present the results of the first systematic study aimed to analyse the genome-wide effects of IR on mutation induction in the mouse germline.

## Results

### Experimental design

**Figure 1:**
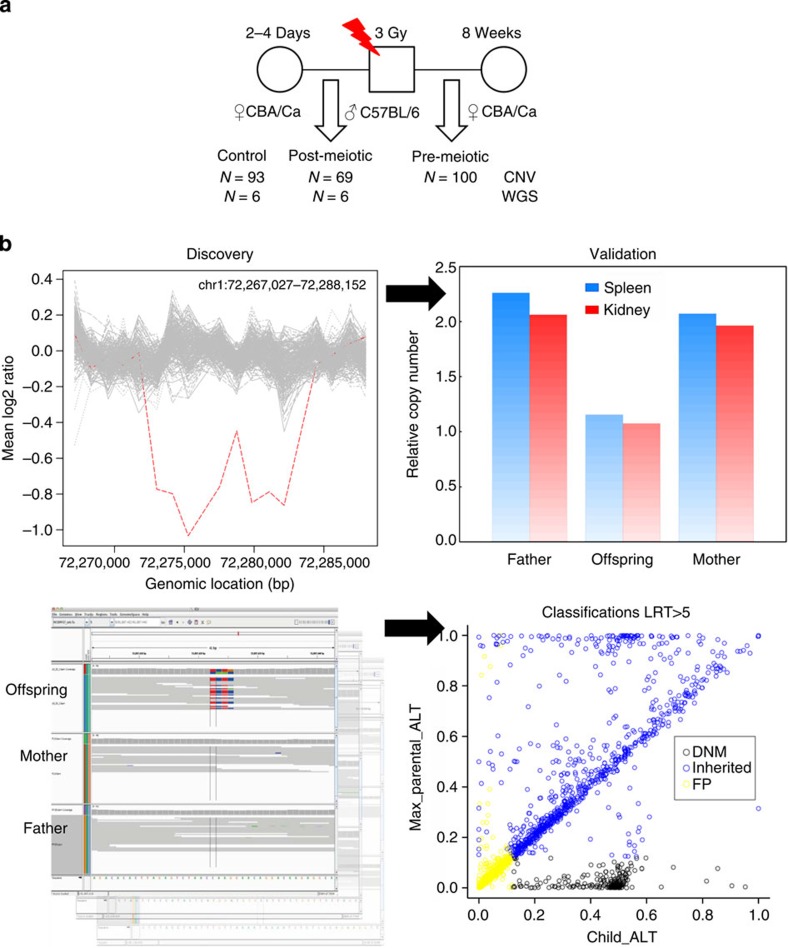
Experimental design and discovery of *de novo* mutations. (**a**) Design of mutational study. The number of offspring analysed by comparative genome hybridization (CNV) and whole-genome sequencing (WGS) is shown. (**b**) Discovery and validation of *de novo* mutations. The panel depicts a representative CNV profile with a 9.1-kb deletion (left) validated by qPCR (right). The bottom panel displays a sequence alignment and discovery of a multisite variant in one offspring (left), and validation of all putative *de novo* SNVs and indels in all offspring (right), where variants are classified as true *de novo* variants, inherited, or false positive categories based on read count proportions of the alternative allele.

We carried out a matched case–control experiment to investigate the immediate and long-term effects of IR on germline mutation in mice. Fifteen C57BL6/J male mice were irradiated at 8-week old, and then mated with two different CBA/Ca females 3 days and then 8 weeks post irradiation (Fig. 1a), thus enabling us to study mutation induction at post-meiotic and pre-meiotic stages of spermatogenesis, respectively^4^. To establish the rate of *de novo* genomic rearrangements resulting in copy number variation in the germline, we used microarray-based comparative genomic hybridization to compare the copy number of case and control offspring and their parents genome-wide with a resolution of ~5.5 kb (Methods). All *de novo* CNVs were validated by quantitative PCR (qPCR; Fig. 1b). In addition, we carried out whole-genome HiSeq sequencing (>22X coverage) on four matched exposed and control pedigrees (Fig. 1a) from matings at the post-meiotic stage in order to capture induced *de novo* SNVs and indels (<50 bp) in the offspring derived from un-repaired radiation-damaged sperm (Fig. 1b). To capture unusual or clustered mutations that may be under-ascertained using standard analytical pipelines, we used several different mutation callers and parameters to call *de novo SNVs* and indels in control and exposed offspring (Methods). We minimized false positives in our analyses by focusing on the non-repetitive portion of the mouse genome, leaving on average, 89.1% of the mappable genome for analysis. After discovery, putative *de novo* SNV and indel candidates were validated using longer read sequencing (Miseq) of PCR amplicons allowing definitive classification of 96% of candidate sites.

### *De novo* CNV mutations

**Figure 2:**
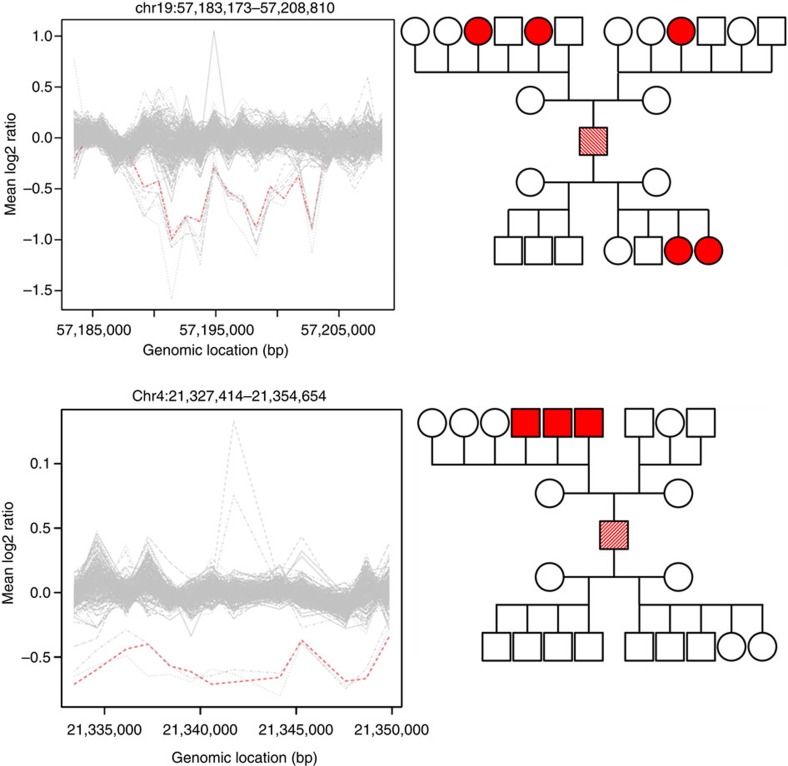
Examples of mosaic *de novo* CNV mutations. Panel on the left represents CNV plots; panel on the right shows segregation of mosaic mutations (in red). The carriers of mosaic mutation are show in hatched red.

The frequency per offspring of *de novo* CNVs in control families is similar to that reported in the previous work^6^. It should be noted that in the offspring of irradiated males we also found four instances of germline mosaicism where the same *de novo* CNV mutation was detected in two or more offspring (Fig. 2). Given that recent exposure to IR during adulthood cannot result in accumulation of mosaic mutations in the paternal germline, these were regarded as spontaneous mutations and excluded from the estimates of mutation rate but used for the analysis of CNV spectra. Our data are in line with the results of recent study showing that mosaicism among parents of children with CNVs is quite common^7^.

**Figure 3:**
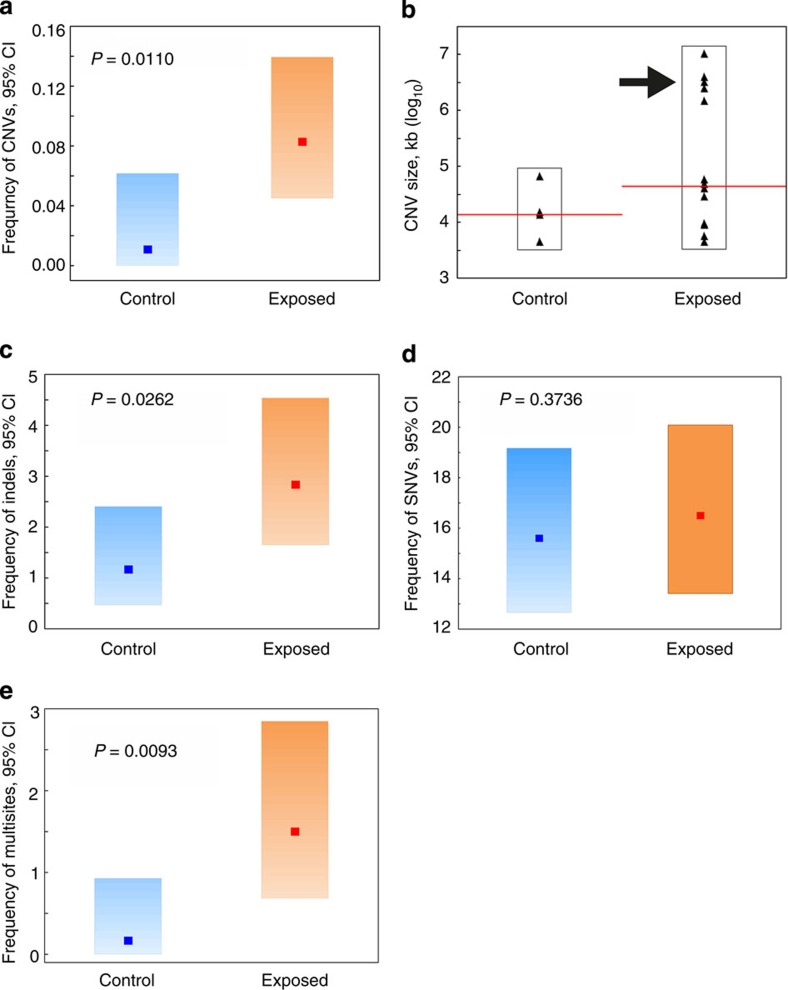
*De novo* mutation frequencies and spectrum. (**a**) Frequency of *de novo* CNV mutations in the offspring of control and irradiated males. (**b**) Variability plot showing the spectrum of *de novo* CNV mutations in the offspring of control and irradiated males (*P*=0.1533, Kruskal–Wallis test). The median values are shown in red; a group of very large CNVs found in the offspring of irradiated males (>1,000 kb) is arrowed. (**c**) Frequency of *de novo* indel mutations in the offspring of control and irradiated males. (**d**) Frequency of *de novo* SNV mutations in the offspring of control and irradiated males. (**e**) Frequency of *de novo* multisite mutations in the offspring of control and irradiated males. The frequency of *de novo* mutations per offspring and probability of difference from controls is shown on all graphs. For CNVs the probabilities were estimated using Fisher’s exact test; for SNVs, indels and multisites, the probabilities were estimated using a one-tailed test based on Poisson simulation of mutations in exposed and control populations (Methods). CI, confidence interval.

We found a significant eightfold increase in the frequency of *de novo* CNVs in the offspring from exposed fathers conceived both pre-meiotically and post-meiotically compared with control offspring (Fig. 3a and Table 1). We could determine the parental origin for ten *de novo* CNVs in cases, with all arising in the paternal germline (Supplementary Table 1), demonstrating increased *de novo* CNV induction by paternal exposure to IR. Using the frequency of *de novo* CNVs found in the offspring of control and irradiated parents, we estimated the doubling dose for CNV mutation as 0.45 Gy (95% CI=0–2.62 Gy), a value close to those obtained in mice using traditional mutation scoring systems^8^. Among the 14 unique germline CNV mutations found in the offspring of irradiated males, 12 were deletions and other 2 were duplications (Supplementary Table 1). We found that the spectrum of radiation-induced CNVs is enriched for very large deletions (>1,000 kb) with the loss of up to 80 genes, mostly protein-coding (Supplementary Data 1 and Fig. 3b), which could potentially be deleterious (Fig. 3b).

### *De novo* SNV and indel mutations

We found that the germline mutation rate for SNVs in mice is less than half that of humans^9^, with a mean across all control offspring of 3.75 × 10^−9^ per nucleotide per generation. Similarly, the indel mutation rate in control offspring is also lower than in humans^9^, although the small numbers preclude a more quantitative evaluation (Supplementary Table 2). Through interrogation of nearby informative heterozygous sites, we could ascertain the parental origin for 27 SNVs in controls, and showed a ratio of 20:7 paternal/maternal origin, which is similar to the established ratio in humans^10^. The structure of shared haplotypes among different lines of inbred laboratory mice^11^ prohibits the phasing of many *de novo* SNVs.

**Figure 4:**
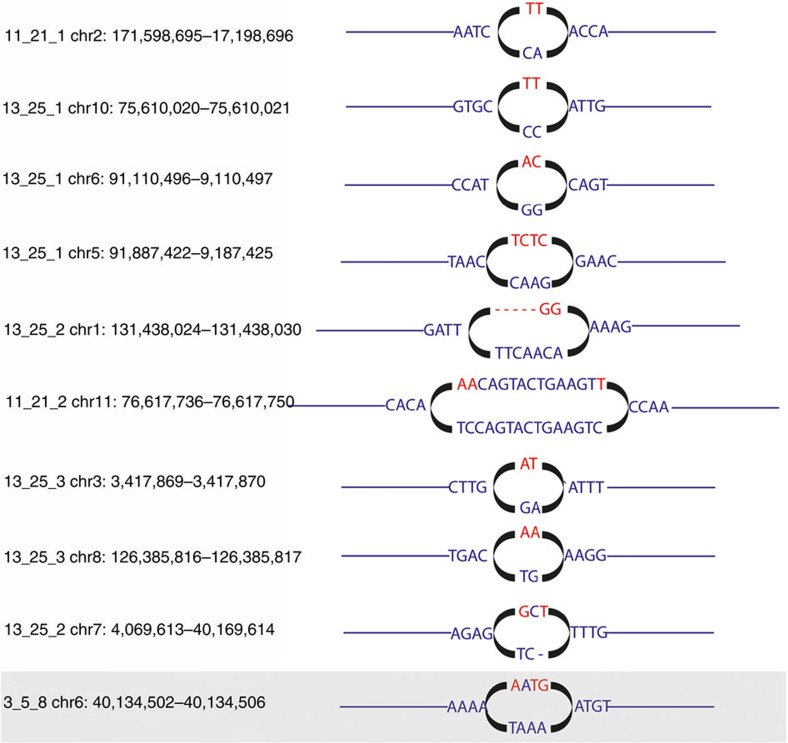
All validated multisite *de novo* mutations found in this study. The reference sequence is shown in blue, mutant in red. Deletions are shown as dashed lines. The final site (boxed) was found in a control sample.

We found that the rate of induction of *de novo* indels was significantly increased, by a factor of 2.4, in offspring of irradiated males (Fig. 3c, Table 1), compared with controls. In contrast, the number of *de novo* SNVs in offspring of exposed fathers is not significantly elevated compared with control offspring (Fig. 3d, Table 1). Although the baseline rate of *de novo* SNVs in the germline of irradiated parents does not significantly exceed that in controls, the spectrum of mutation is markedly different. Specifically, we found the frequency of clustered *de novo* mutations (clusters of 1–4 SNVs or clusters of 1–2 SNVs and indels within a few base-pairs of each other, Fig. 4), to be significantly elevated in the offspring of irradiated fathers compared with controls (Fig. 3e and Table 1). Although the presence of a single occurrence of clustered mutation in a control offspring supports the notion that clustered mutations occur at a low level in unexposed individuals^12^,^13^, the enrichment of clustered mutations in the offspring of exposed males can be attributed to the induction of clustered damaged sites in the germline of irradiated males. Clustered DNA-damaged sites, defined as two or more lesions within one or two helical turns of the DNA, are regarded as a signature of radiation exposure^14^,^15^. Judging from the results on the induction of clustered DNA damage sites in irradiated mammalian cells^16^, exposure to 3 Gy of X-rays may induce up to 1,000 multiply damaged sites in the germline of irradiated male mice. Given that the efficiency of their repair is substantially compromised and delayed^15^,^16^, radiation-induced clustered damaged sites can lead to multiple nucleotide substitutions occurring within short stretches of DNA. It should be noted that as DNA repair in mature sperm is effectively shut down, all DNA damaged sites attributed to post-meiotic exposure are recognized and repaired after fertilization^17^. None of the spontaneous or induced *de novo* SNVs, indels or multisites are predicted to have had any phenotypic impact on the offspring; only two SNVs and two indels fall in exonic regions (in genes *Adams20*, *Tmed9*, *Cep250* and *H2afy*), knockouts of which show no phenotypic changes.

## Conclusions

**Figure 5:**
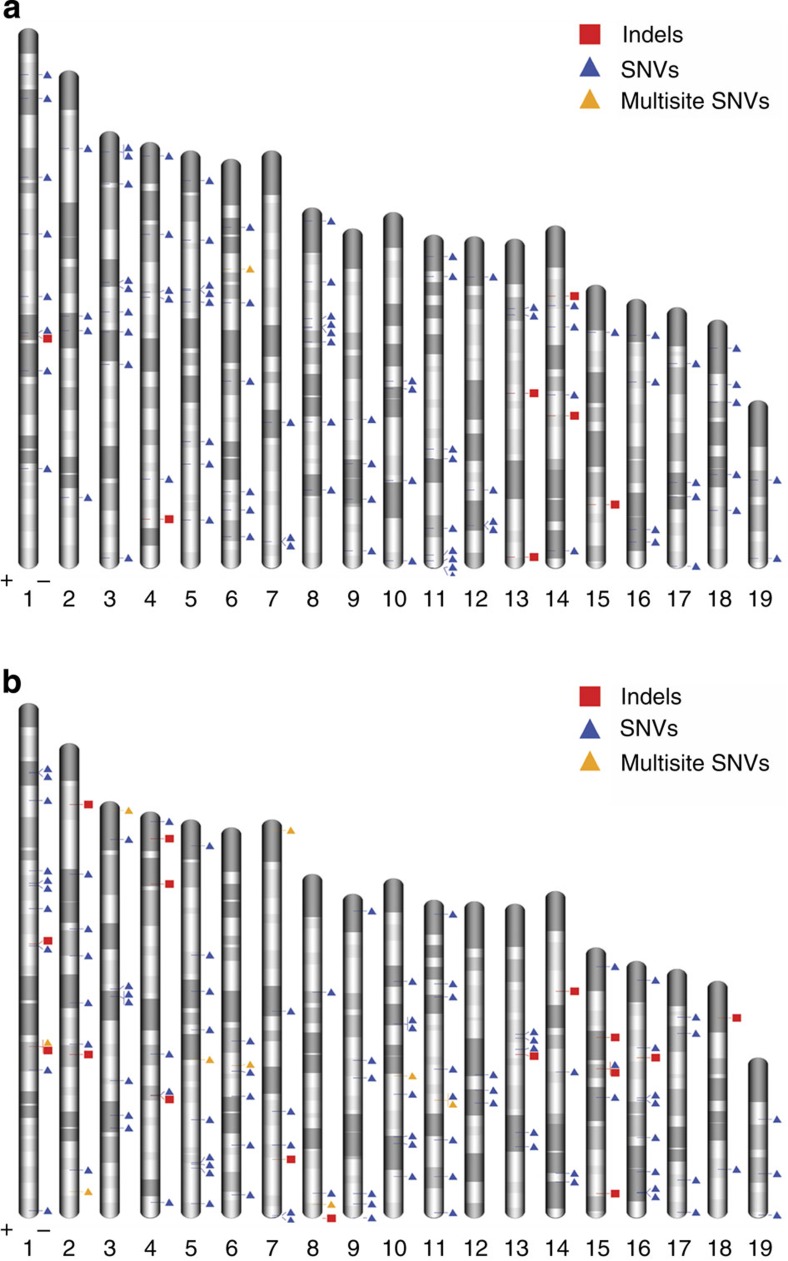
Chromosomal distribution of *de novo* mutations found in this study. SNVs, CNVs, indels and multisite variants are shown for offspring that were whole-genome sequenced. The six control (**a**) and six exposed (**b**) offspring are shown on one plot, respectively. Plots drawn by Idiographica^28^.

In summary, we have validated the use of whole-genome approaches for characterizing the signatures of mutagen exposure in the mammalian germline and we report a systematic evaluation of the genome-wide effects of irradiation on mutation induction. Our *in vivo* genome-wide study differs from previous work, which has focused on either specific loci, or limited to bacterial cells and cell lines. We observed an increased magnitude of mutation induction with increasing size of the mutation event; paternal irradiation significantly increased the incidence of large (>5.5 kb) CNVs, small (<50 bp) indels and multiple SNVs, whereas the total frequency of *de novo* SNV mutations among the offspring of exposed males was not significantly elevated. While we did not screen for intermediate sized 50 bp to 5.5 kb structural variants, it is reasonable to expect this class of variation would also show significant mutation induction by IR given the shared mutational mechanisms with the larger (CNVs) and smaller (indel) mutation classes that are significantly induced. In our study, we did not observe any stratification in numbers or types of *de novo* mutation in the sex of the offspring, or any significant clustering of induced or spontaneous mutations in or between offspring (Fig. 5).

The genome-wide magnitude of mutation induction of SNVs that we observed may appear to be less than previously thought. It should be noted that the vast majority of radiation-induced DNA damage is attributed to singly damaged bases^1^. According to the data from ref. 1, exposure to 3 Gy of acute X-rays should induce up to 6,000 damaged bases in the germline of irradiated males. Given that a diploid mammalian cell repairs 30,000 similar endogenous DNA lesions per day^18^, the induction of an extra 6,000 DNA lesions should not represent a sufficient challenge to the DNA repair machinery. In contrast, radiation-induced double-strand breaks and clustered damaged sites constitute the most dangerous types of DNA damage, as their repair is substantially delayed or compromised^15^,^16^. If not adequately repaired, these lesions would cause DNA rearrangements, including CNVs and indels, as well as clustered nucleotide substitutions, thus creating a specific signature of radiation exposure, described by our whole-genome study. We note that a signature of clustered mutations on exposure of *Caenorhabditis elegans* to chemical mutagens was recently described by Meier and colleagues^19^. Applying the strategy adopted here to characterize germline mutation induction across a wide range of mutagenic exposures should lead to a step change in our understanding of the sensitivity of the mammalian germline to exogenous mutagens.

## Methods

### Mice

C57BL/6 (male) and CBA/Ca (female) mice were obtained from Harlan. Eight-week-old male mice were given whole-body acute irradiation of 3 Gy of X-rays delivered at 0.5 Gy min^−1^ (250 kV constant potential, HLV 1.5 mm Cu, Pantak industrial X-ray machine). Irradiated and sham-treated male mice were each mated with two age-matched non-irradiated CBA/Ca females either for 4 days immediately or 8 weeks post exposure. This design allows us to profile the effect of IR at two distinct stages of spermatogenesis. We referred to these stages as post-meiotic (mating immediately after irradiation) and pre-meiotic (mating after 8 weeks post-irradiation). Tissue samples were taken from all the parents and their 8-week-old offspring. The study protocol was approved by the University of Leicester Research Ethical Review Committee and performed under the Home Office project licence No. PPL 80/2267.

### Microarray analysis

Genomic DNA was extracted from spleen and kidney by phenol-chloroform following standard protocols with 1% SDS and 300 μg ml^−1^ proteinase K (Roche Diagnostics Limited). DNA was quantified using ultraviolet spectroscopy (NanoDrop 1000, Thermo Scientific) and the quality checked on 1% agarose gel. For the comparative genomic hybridization, spleen DNA was sent to Roche NimbleGen Service Laboratory (Reykjavík, Iceland) for full service (sample labelling, hybridization and scanning). The labelled samples were hybridized to mouse ultra-high-resolution Comparative Genome Hybridization (CGH) arrays (Mouse 2.1M CGH, 080411_MM9_CGH_HX1, NimbleGen, Roche Diagnostics) with 2.1 million features and 1.1 kb median probe spacing. An equal molar dilution of father and mother genomic DNA was used as the reference genome for each offspring.

### Quality control for hybridization data

The quality of hybridization data received from NimbleGen service centre was examined using two approaches. First, by generating a spatial plot for each of the array data and visually checking or any artefacts from the hybridization or inconsistencies between/ or within arrays. The plots were generated using Ringo package^20^ in R environment. From this QC measure, four of the 262 arrays failed owning to hybridization artefacts. The second approach employed median absolute deviation (MAD) of log2 ratios as the measure of noise in the data. The threshold for accepting array data was set at MAD>0.16 based on the distribution of the MAD values across the whole samples. Only array data that passed these QC procedures was used in calling CNVs.

### Array based CNV discovery

The copy number calling was done using circular binary segmentation algorithm in R bioconductor DNAcopy package with additional post-quality control steps. Before the CNVs calling, the q-spline normalized data from NimbleGen was corrected for wave effect using an in-house algorithm. The threshold for accepting a CNV was set to comprise at least five probes with a median log2 ratio±0.40. Sex chromosomes were not included in the analysis.

### Validation of CNVs

Real-time qPCR was used to validate the relative copy number of CNVs detected by array-CGH in two tissues: spleen and kidney. All qPCR experiments were performed on LightCycler 480 System (Roche Applied Science) using TaqMan detection chemistry. Mouse *Transferrin receptor protein 1* (*Tfrc1*) was chosen as a reference target owning to its copy number consistency across our array-CGH data. Primers and probes were designed with Beacon Designer Free Edition (Premier Biosoft International) to accommodate duplexing TaqMan qPCR (primer and probe sequences provided in Supplementary Table 3). The reference probe was labelled with VIC dye at the 5′ end and minor groove binder quencher at the 3′ end. All the target probes were labelled with FAM dye and minor groove binder quencher at the 3′ end according to the manufacturer’s protocol (Life Technologies). To ensure similar amplification efficiency between the target and the reference primers, standard curves were generated for each target and reference and only those in the range of 1.95–2.10 were accepted. Each qPCR assay was performed in quadruplicate and in duplex (a target and reference target *Tfrc1*). Each reaction contained 10 ng of genomic DNA, 2 × of SensiMix II probe (Bioline, UK), 0.1 μM of target probe, 0.1 μM of *Tfrc1* probe, 0.5 μM each of forward and reverse primers for the target and *Trfrc1*, respectively, in a total reaction volume of 5 μl. The qPCR thermal cycling conditions were as follows: initiation at 95 °C for 10 min for hot start, followed by 45 cycles of 95 °C for 10 s and 56 °C for 1 min. Data analysis was further performed using the 2^−ΔΔ*C*^_T_ method^21^.

### Determining parental genotypes

The haplotype of parents and offspring with CNVs was determined by Sanger sequencing. Briefly, primers were designed to amplify a genomic region within the CNVs (deletions) by PCR. The PCR products were purified using the Zymoclean Gel DNA recovery kit (Zymo Research) and sequenced. The DNA sequences were aligned using Multiple Sequence Comparison by Log-Expectation^22^ to check for single-nucleotide polymorphisms (SNPs), which varied between the parents. The pattern of these SNPs was examined in the offspring with deleted CNV under consideration. Since we profiled first generation mice for CNVs, we expect to see heterozygotes for any given polymorphism, therefore loss of heterozyogsity enable us to trace the parental origin of the CNV.

### Sequencing

DNA extracted from the spleen of parents and their offspring was pooled and sequenced using standard protocols and Illumina HiSeq technologies to an average depth of 25X. The resultant sequence data were aligned to mouse reference NCBI37. Duplicate reads were removed, and BAM improvement carried out at lane and sample level according to GATK best practice^23^ before lane and sample data were merged. The total mapped sequence coverage was 23.3, 24.6, 30.4, 24.1, 24.2, 25.5 reads per base for the offspring from irradiated fathers (11_21_1, 11_21_2, 11_21_3, 13_25_1, 13_25_2, 13_25_3), and 26, 26.4, 26.1, 23.1, 23.9, 29.2 reads per base for the control offspring (2_3_1, 2_3_2, 2_3_3, 3_5_1, 3_5_4, 3_5_8). The mapped reads per based for the parents were 23.4, 24.4 for the irradiated fathers (P11, P13), 25.8 and 24.1 for the mothers from the irradiated trios (PF21, PF25), and finally 30.6 and 22.5 mapped reads per base for the control mothers (PF3, PF5).

### Variant calling

Variants were called using bcftools^24^ and samtools^24^, and standard settings. Variants were also called using samtools without BAQ alignment, although no additional candidates were added to the validation data set from this analysis.

### *De novo* mutation calling

*De novo* mutations were called on the variants supplied by bcftools using *DeNovo*Gear version 0.5 (ref. 25) and Triodenovo (http://genome.sph.umich.edu/wiki/Triodenovo) using standard settings. DeNovoGear called 3,303, 3,284, 4,245, 3,500, 3,593 and 3,480 SNVs and small indel candidates in the autosomes of the offspring from irradiated fathers (11_21_1, 11_21_2, 11_21_3, 13_25_1, 13_25_2, 13_25_3, respectively) and 4,015, 4,334, 4,022, 3,172, 3,737 and 3,919 candidates in autosomes from the control offspring (2_3_1, 2_3_2, 2_3_3, 3_5_1, 3_5_4, 3_5_8, respectively). Calls from the X chromosome were discarded as SNVs and indels showed a gender-associated inflation, which was not possible to correct for. DeNovoGear was re-run with a tenfold lower posterior probability threshold to capture low-quality indels and clustered SNVs.

### Filtering

Candidate *de novo* variants were filtered to exclude simple sequence repeats and segmental duplications where we expected false positives to be enriched (on average 26–30% of candidates fall in a segmental duplication and 23–27% in a simple sequence repeat, although these categories are not exclusive of each other). Assuming a Poisson distribution for sequencing depth, sites with a depth greater than the 0.0001 quantile were removed due to the likelihood of mapping errors or low complexity repeats introducing false positives (generally 77–80% of candidate sites). Candidate sites where the *de novo* allele was present in either parent in greater than 0.05% of reads and where there were known SNPs in the parental strain were also removed on the grounds that they were likely to be inherited (4.06–6.21% and 0–4.73% of candidate sites, respectively). Once these filters were applied, 172, 156, 157, 183, 160 and 149 indel and SNVs candidate *de novos* remained for validation in the offspring from irradiated fathers (11_21_1, 11_21_2, 11_21_3, 13_25_1, 13_25_2 and 13_25_3), along with 174, 183, 168, 166, 172 and 149 small indel and SNVs candidate *de novos* for control offspring (2_3_1, 2_3_2, 2_3_3, 3_5_1, 3_5_4, 3_5_8).

Given the potential diverse nature of the mutations that may be induced in the offspring from irradiated fathers, we tried three different strategies to increase the numbers of candidate variants before validation. First, we tried a different *de novo* variant caller (Triodenovo) to call *de novo* mutations in all trios, using our standard variant calls. Visual inspection showed that most high-quality candidate calls were called by both callers. However, 1,171 sites that were called by Triodenovo and not by DeNovoGear across all 12 trios were manually reviewed, leading to 46 plausible *de novo* candidates. After filtering these 46 Triodenovo calls using the same criteria as above, 13 candidates remained that were added to the validation callset (four calls in 2_3_1, three calls in 11_21_1, three calls in 3_5_1 and one call each in 11_21_3, 13_35_3 and 3_5_8). Second, we re-ran DeNovoGear using standard variant calls but decreased the posterior probability threshold by a factor of 10 to allow for more low-quality indel *de novo* calls. After filtering as above, 811 sites were manually reviewed and 46 were added to the callset for validation. (5, 4, 3, 5, 4 and 3 sites from offspring from irradiated fathers and 5, 2, 6, 4, 3, 3 from offspring from control fathers, respectively). Last, we re-ran Samtools without BAQ re-alignment in order to increase the number of clustered mutations called by Samtools. Candidate sites were filtered as before and any previously considered candidates were removed. This led to 120–200 additional candidates’ sites per trio where there were two candidate sites directly adjacent to each other. A proportion of these sites were manually reviewed but no further candidates were added to the validation callset. In total, 2,048 candidate *de novo* mutations were put forward for validation (Supplementary Table 4). We intentionally included many low-confidence calls in our validation callset in order to maximize sensitivity.

### Experimental validation

A sequence of 400 bp flanking the putative mutation were extracted, and primers were designed using Primer 3 (ref. 26) so that the mutation lay in the centre of a 300- to 425-bp PCR product. Primers were designed to have a GC clamp, be optimally 20 bp in length and with a melting temperature of 60°. All primers were ordered from Sigma Genosys. Using this strategy, 2,034/2,048 regions resulted in successful primer design. The region-specific primers were then tailed with a standard forward and reverse sequencing primer.

Forward sequencing primer 5′-ACACTCTTTCCCTACACGACGCTCTTCCGATCT-3′

Reverse sequencing primer 5′-CGGTCTCGGCATTCCTGCTGAACCGCTCTTCCGATCT-3′

These primers were used to generate PCR products using RedAccuLATaq from Sigma Aldrich, 10 μM primer and a touchdown PCR protocol. Each sample was then tagged with Illumina primers and a primer specific to each individual in a second round of PCR, using biotyinlated primers, Kapa HiFi Taq (Kapa Biosystems) Taq and 5 μl of first round PCR product as template.

The second round PCR products were pooled by individual and then cleaned up using Agencourt Ampure SPRI beads from Beckman Coulter. All individuals were then pooled together, and the pool balances checked. Three lanes of the balanced pools were run on an Illumina Miseq platform (250 bp paired end reads), leading to an average of 100X coverage across the candidate site of interest. The resultant sequence data were merged by individual and annotated with read counts at the candidate site using an in house python script. An in house R script (http://www.R-project.org) was then used to allocate a likelihood to each candidate variant being a true *de novo* mutation, an inherited variant or a false positive call, based on the allele counts of the parents and child at that locus. A proportion of the SNV candidates as well as all of the indel candidates were reviewed manually using Integrative Genomics Viewer (IGV). A summary of validation is given in Supplementary Table 5.

### Haplotyping of *de novo* SNVs and indels

We used the read-pair algorithm supplied with the DeNovogear software to determine the parent of origin of our validated *de novo* mutations using the deep whole-genome sequence data. DeNovoGear uses information from flanking variants that are not shared between parents to calculate the haplotype on which the mutation arose. Using this technique, we were able to confidently assign the parental haplotype in 49/224 validated *de novo* mutations.

### Mutation rate estimation

We calculated a mutation rate for autosomal SNVs in each individual as follows: first, we calculated the proportion of the genome not covered in our analysis because of the depth of the whole-genome sequencing: Bedtools^27^ was used to calculate the proportion of the genome not considered in our analysis due to low- or high-sequence depths for each individual (mean 8.4%). We then calculated what proportion of sites were removed by our whole-genome filters (simple sequence repeats and segmental duplications) after the depth filters were applied (average 2.7%). Last, we used the posterior probability supplied by DeNovoGear to calculate what proportion of sites that were not validated (failed validation or removed by to filters), would be likely to be true *de novo* mutations.

### Calculation of rate significance

We assessed the significance of the difference in numbers of SNV, multisite and indel mutations between offspring of exposed and control fathers using a one-tailed test based on Poisson simulation of the null distribution of the difference in the numbers of mutations observed in offspring of exposed and control fathers, assuming the same mutation rate across exposed and control offspring based on the average mutation rate across the two groups. Significance was estimated by calculating the proportion of the 100,000 simulations that gave an excess of numbers of mutations in case offspring compared with control offspring that was the same or greater than observed (Supplementary Fig. 1).

### Functional annotation of validated *de novo* variants

Annotation of SNVs, indels and multisite variants were carried out using ANNOVAR (ref. 28).

## Author contributions

Y.E.D. and M.E.H. supervised the project, generated the original hypothesis and designed the study. A.B.A and S.J.L. performed the experiments. A.B.A, S.J.L., Y.E.D. and M.E.H. analysed the data and wrote the manuscript. All authors read and approved the final manuscript.

## Additional information

**Accession codes**: The microarray data were deposited in the Gene Expression Omnibus (GEO), submission number GSE65521; the whole-genome sequences were deposited in the Sequence Read Archive under Study number ERP001221, with accession numbers ERX111346, ERX111347, ERX111348, ERX111349, ERX111350, ERX111351, ERX111352, ERX111353, ERX111354, ERX111355, ERX111356, ERX111357, ERX111358, ERX111359, ERX111360, ERX111361, ERX111362, ERX111363, ERX111364, ERX111365, ERX111366 and ERX111367.

**How to cite this article:** Adewoye, A. B. *et al*. The genome-wide effects of ionizing radiation on mutation induction in the mammalian germline. *Nat. Commun.* 6:6684 doi: 10.1038/ncomms7684 (2015).

## Supplementary Material

Supplementary Figure and Supplementary Tables Supplementary Figure 1 and Supplementary Tables 1-5

Supplementary Data 1 Structure of de novo CNV mutations

## Figures and Tables

**Table 1:** *De novo* mutation frequencies in the offspring of control and irradiated parents.

**Group**	No mutations*	**Mutation frequency (95% CI)**	**Ratio**†	**Probability**‡
De novo *CNV*				
Control	1 (93)	0.0108 (4.30 × 10^−6^–0.0616)	—	—
Post-meiotic	5 (69)	0.0725 (0.0229–0.1705)	6.74	0.0510
Pre-meiotic	9 (100)	0.0900 (0.0408–0.1716)	8.37	0.0125
All irradiated	14 (169)	0.0828 (0.0451–0.1394)	7.70	0.0110
*Indels*				
Control	7 (6)	1.1667 (0.4690–2.4037)	—	—
Post-meiotic	17 (6)	2.8333 (1.6505–4.5364)	2.43	0.0262
*SNVs*				
Control	94 (6)	15.6000 (12.6602–19.1720)	—	—
Post-meiotic	99 (6)	16.5000 (13.4104–20.0882)	1.05	0.3736
				
*Multisite*				
Control	1 (6)	0.1667 (0.0040–0.9286)	—	—
Post-meiotic	9 (6)	1.5000 (0.6859–2.8474)	9.00	0.0093

CI, confidence interval; CNV, copy number variant; Indels, insertion/deletion events; SNV, single-nucleotide variant.

^*^The number of offspring is given in brackets.

^†^Ratio to the frequency of *de novo* mutations in controls.

^‡^Probability of difference from controls. For all the *de novo* CNV mutations the probability was estimated using Fisher’s exact test, one-tailed, and for the remaining classes of mutation using a one-tailed test based on Poisson simulation of mutations in exposed and control populations (Methods).
