# Supplementary material for: The genome-wide effects of ionizing radiation on mutation induction in the mammalian germline
Source: Nat Commun. 2015 Mar 26;6:6684. doi: 10.1038/ncomms7684 (PMC4389250; doi:10.1038/ncomms7684)
Supplement: Supplementary Figure and Supplementary Tables — Supplementary Figure 1 and Supplementary Tables 1-5 [file ncomms7684-s1.pdf]

Supplementary Fig. 1

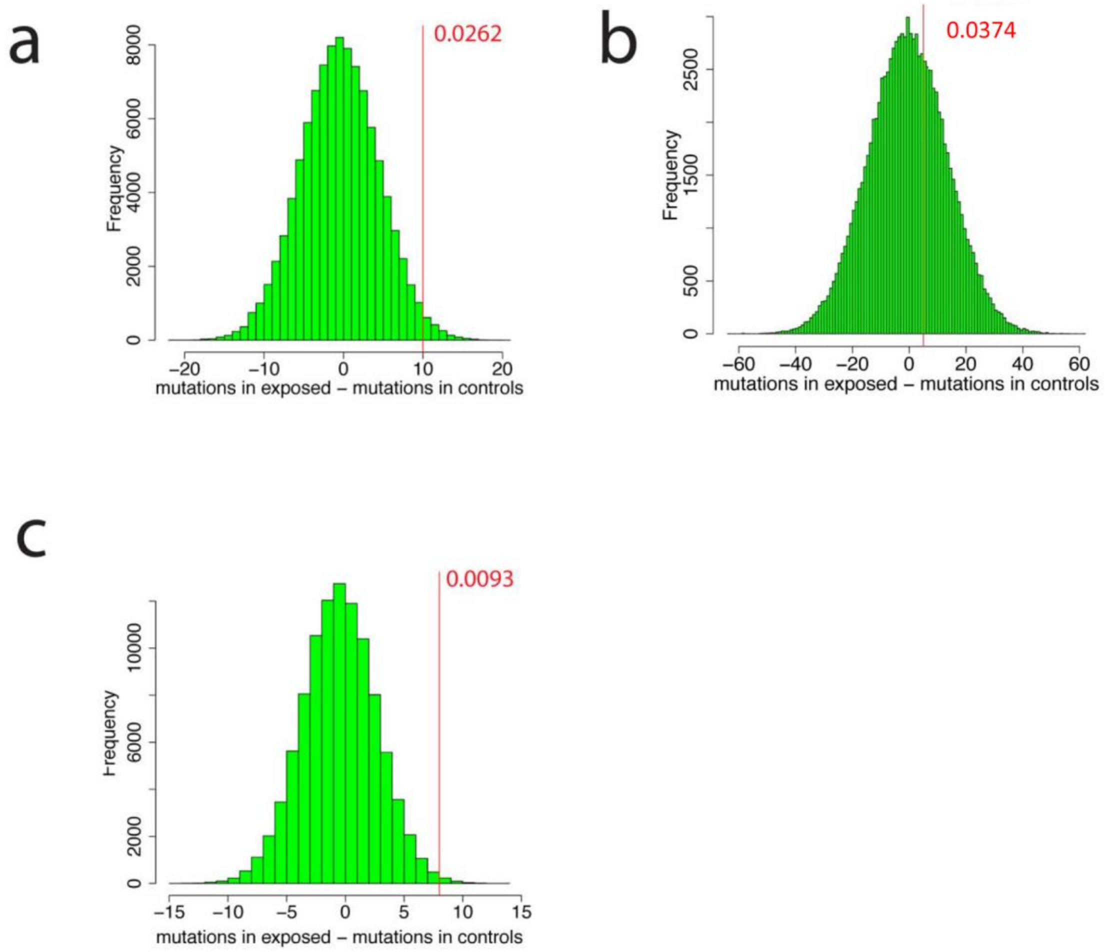

Supplementary Figure 1 | Histograms showing Poisson simulations of the null distribution of the difference in numbers of *de novo* mutations in exposed and control populations of mice. The red line shows the true difference between numbers of mutations and the chances of this occurring over 100,000 simulations, in indels (a), SNVs (b), and multisite (c*) de novo* mutations.

Supplementary Table 1 | Spectrum of *de novo* CNV mutations.

ID	Chrom	Start	End	Size	Sex*	Type	Origin	Comment†
Control								
CNVc1	11	25,905,881	25,910,330	4,449	♂	del	?	unique
Irradiated, post-meiotic								
CNVa8	4	21,333,414	21,348,654	15,240	-	del	?	mosaic (3)
CNVa1	1	7,897,894	11,887,864	3,989,970	♂	del	pat	unique
CNVa13	5	65,687,317	65,693,004	5,687	♀	del	?	unique
CNVa7	8	18,705,709	18,764,523	58,814	♀	del	pat	unique
CNVab12‡	19	57,189,173	57,202,810	13,637	-	del	pat	mosaic (2)
CNVa36	16	25,331,300	25,371,606	40,306	♂	dupl	?	unique
CNVa15	5	78,795,493	89,129,706	10,334,213	♂	del	pat	unique
Irradiated, pre-meiotic								
CNVb35	13	76,293,133	79,528,307	3,235,174	♀	del	pat	unique
CNVb54	12	100,826,223	100,830,676	4,453	-	dupl	pat	mosaic (2)
CNVb14	16	40,041,302	40,045,796	4,494	♂	del	pat	unique
CNVb16	11	41,634,027	41,638,541	4,514	♂	dupl	?	unique
CNVb32	1	72,273,027	72,282,152	9,125	♂	del	?	unique
CNVb6	14	15,306,083	17,774,817	2,468,734	♀	del	pat	unique
CNVb28	15	61,779,112	61,788,603	9,491	♀	del	pat	unique
CNVb31	16	72,137,165	72,166,552	29,387	♂	del	pat	unique
CNVb18	4	48,968,827	49,017,041	48,214	♂	del	pat	unique
CNVb57	14	114,841,926	116,324,868	1,482,942	♀	del	pat	unique
CNVb15	16	41,628,748	41,695,564	66,816	♀	del	pat	mosaic (2)
CNVab12‡	19	57,189,173	57,202,810	13,637	-	del	pat	mosaic (3)

*Sex of offspring

†The number of offspring sharing mosaic mutations is given in brackets.

‡The same *de novo* mosaic mutation detected in the offspring of one irradiated male.

Supplementary Table 2 | *De novo* mutations detected by the next generation sequencing.

Group, ID	Indel*	SNV*	Multisite	False positive	Inherited	NA†
**Control**						
2_3_1	0	19	0	64	93	6
2_3_2	1	15	0	75	90	5
2_3_3	3	22	0	91	49	8
3_5_1	1	17	0	101	49	4
3_5_4	0	13	0	83	66	13
3_5_8	2	9	1	66	68	6
**Total**	**7**	**95**	**1**	**480**	**415**	**42**
**Irradiated**						
11_21_1	5	18	1	76	78	4
11_21_2	3	21	1	64	60	11
11_21_3	2	14	0	56	82	6
13_25_1	1	24	3	57	89	12
13_25_2	2	19	2	66	75	2
13_23_3	4	11	2	62	73	1
**Total**	**17**	**107**	**9**	**381**	**457**	**36**

*Multisite mutations counted as one mutational event.

†Including primer design and validation fail.

Supplementary Table 3 | Primers used for de novo CNV validation by qPCR.

Name	Primers (forward/reverse)	Chrom.	Start	End
Tfrc1	GCACTAGATTGGATACCTA<br>ACGTCATGGGTAAGTTTA	16	32,626,657	32,626,771
CNVc1	GGCTTTCCATTCTGAATC<br>TGGTGTTACTTAGGATCA	11	25,806,686	25,806,827
CNVa8	CCAGAACTGATCAAAAGAA<br>CTGGTAAGTAGAACACAAC	4	21,267,392	21,267,496
CNVa2				
-Region 1	CCTACTCCAAGAAGACAA<br>GGATCAGAAAGTGGAATG	1	9,524,462	9,524,577
-Region 2	GGCTGGTTATTGGAAAAG<br>AGGCTTATTAAACTTGATTTC	1	11,582,349	11,582,448
CNVa13_1	GTAGGCAGGTATTTGAAG<br>AGCACAAAGTAAATTCACA	5	66,079,681	66,079,790
CNVa7	TCCTCTGGCTCTATGATA<br>CAGTCTGTTTATACTTTTACATG	8	18,706,670	18,706,801
CNVa15				
-Region 1	CCACACAATACACACAATA<br>CCCACTTTTCTAGTTGATG	5	79,224,965	79,225,052
-Region 2	GTGCTTTGAGCTTTACTG<br>GGACACTTCATACTCTTCA	5	79,000,502	79,000,600
-Region 3	AGGGAAATACTTTTCAACTC<br>TGGGTAACTGTGAATAATATC	5	79,724,211	79,724,302
-Region 4	GCCAATAAGTGACTGATC<br>CATGCTCCTGTCTTTATG	5	80,340,545	80,340,644
CNVb35				
-Region 1	CTCCATCACAGCAAGAATA<br>CATCCATGACACTGTCTTA	13	76,431,209	76,431,357
-Region 2	GCTTGTAACAATATGTGTG<br>GCTGCTTTTGATGATAGTA	13	77,629,724	77,629,853
CNVb14	GGACTCATAGGCATTCTA<br>GAGTGTCTATATCTGTCTGA	16	40,042,318	40,042,435
CNVb16	GAGAGTAACATGATCATTTACA<br>GCTGATTAGTTTCAATCTGTA	11	41,448,496	41,448,578
CNVb32	CTCAGTTACTTGGCAGAA<br>AGGTGTACACTTTTGTCA	1	72,320,313	72,320,387
CNVb6				
-Region 1	CCTTCAGAATTACGTCATTA<br>CCCTCATAGAGTCTCTAG	14	16,138,747	16,138,829
-Region 2	CCTCAGCTTCTAGAGATTA<br>CTCAGAAAGGTTTCATTTAGTA	14	16,278,955	16,279,040
CNVb28	GAGGACACAGTATCTTCTA<br>CCCTTTCTTCTTGCTATG	15	61,611,119	61,611,240

Supplementary Table 4 | Candidate sites chosen for validation.

**ID**	**Indel candidates**	**SNV candidates**	**Additional indel candidates***	**Trio *de novo* candidates**	**Total**
**Controls**					
2_3_1	45	129	5	4	**183**
2_3_2	47	136	2	0	**185**
2_3_3	39	129	6	0	**174**
3_5_1	23	143	4	3	**173**
3_5_4	14	158	3	0	**175**
3_5_8	18	131	3	1	**153**
**Irradiated**					
11_21_1	24	148	5	3	**180**
11_21_2	26	130	4	0	**160**
11_21_3	22	135	2	1	**160**
13_25_1	27	156	5	0	**188**
13_25_2	37	123	4	0	**164**
13_25_3	23	126	3	1	**153**
**Total**	345	1644	46	13	**2048**

*Using lowered posterior probability threshold.

Supplementary Table 5 | Validation outcome.

ID	For validation	Passed primer design	Passed validation (LRT>5)	Inherited	False positive	DNM	Adjusted DNM*
**Controls**							
2_3_1	183	183	177	93	64	19	19
2_3_2	185	183	180	90	75	16	16
2_3_3	174	173	166	49	91	25	25
3_5_1	173	173	167	49	101	17	17
3_5_4	175	175	162	66	83	13	13
3_5_8	153	151	147	68	66	13	11
**Irradiated**							
11_21_1	180	178	176	78	76	22	23
11_21_2	160	158	149	60	64	25	24
11_21_3	160	158	154	82	56	16	16
13_25_1	188	187	175	89	57	29	25
13_25_2	164	163	162	75	66	21	21
13_25_3	153	152	152	73	62	17	15
**Total**	2048	2034	1967	872	861	234	225

* Multisite variants counted as one mutational event, includes indels.
